# The Effects of Two Thick Film Deposition Methods on Tin Dioxide Gas Sensor Performance

**DOI:** 10.3390/s90906853

**Published:** 2009-08-31

**Authors:** Smitesh D. Bakrania, Margaret S. Wooldridge

**Affiliations:** 1 Department of Mechanical Engineering, Rowan University / 201 Mullica Hill Road, Glassboro, NJ 08028, USA; 2 Department of Mechanical Engineering, University of Michigan / 2350 Hayward Street, Ann Arbor, MI 48109, USA; E-Mail: mswool@umich.edu

**Keywords:** SnO_2_, gas sensor, fabrication, combustion, deposition, film, binder

## Abstract

This work demonstrates the variability in performance between SnO_2_ thick film gas sensors prepared using two types of film deposition methods. SnO_2_ powders were deposited on sensor platforms with and without the use of binders. Three commonly utilized binder recipes were investigated, and a new binder-less deposition procedure was developed and characterized. The binder recipes yielded sensors with poor film uniformity and poor structural integrity, compared to the binder-less deposition method. Sensor performance at a fixed operating temperature of 330 °C for the different film deposition methods was evaluated by exposure to 500 ppm of the target gas carbon monoxide. A consequence of the poor film structure, large variability and poor signal properties were observed with the sensors fabricated using binders. Specifically, the sensors created using the binder recipes yielded sensor responses that varied widely (e.g., *S* = 5 – 20), often with hysteresis in the sensor signal. Repeatable and high quality performance was observed for the sensors prepared using the binder-less dispersion-drop method with good sensor response upon exposure to 500 ppm CO (*S* = 4.0) at an operating temperature of 330 °C, low standard deviation to the sensor response (±0.35) and no signal hysteresis.

## 1. Introduction

As emissions regulations become more stringent and process controls demand more accurate and diverse information, there is an ever-increasing need for gas sensors with superior performance. For example, carbon monoxide detectors based on tin dioxide (SnO_2_) gas sensors have become a standard recommended for every residence and workplace and represent a dramatic improvement over passive colorimetric sensors which were the prior state of the art. Solid state gas sensors, which use films of metal oxides, such as tin dioxide, zirconia (ZrO), and titania (TiO_2_), as the active sensing materials have long been demonstrated as having sensor response to few parts per million gas concentration and selectivity at low operating temperatures [1–4]. Nevertheless, to design sensors with improved and targeted functionalities, a more fundamental understanding is necessary of how the microstructural film properties, in addition to the inherent material activity, affect gas detection. Establishing links between micro-architecture and bulk performance, as current research indicates, is a challenging problem with interdependent factors governing sensor behavior [5]. An additional layer of complexity is introduced considering the active sensing materials need to function as an electrical component and have good mechanical integrity, and the materials and electronic properties are affected by the sensor fabrication process [6–8]. Improved understanding of the effects of film fabrication on sensor performance is critical to advancing sensor design.

Sensor film fabrication is a complex process that can restrict, limit and define sensor performance. For example, in the case of sputtered films compared to screen printed films, the deposition method directly influences the film density and therefore the baseline resistance of the sensor [9,10]. Binders are commonly employed to deposit tin dioxide films to improve the mechanical strength of the film. Such binders have noticeable interactions with sensor performance, especially when used with organic vehicles [7,11,12]. Along with other factors such as film processing (e.g., heat treating) and film imperfections, the film deposition process itself can modify the sensor response sufficiently to mask the influence of material properties [5,13–16]. The objective of this work was to develop a simplified deposition method that did not require complex instrumentation, such as screen printers and vacuum chambers, yet would also yield repeatable results in terms of sensor performance. Such a method could be used to explore the links between material properties and sensor performance without interference from fabrication effects. The technical approach was to evaluate two categories of deposition methods and the resulting effects on tin dioxide gas sensor performance. Specifically, SnO_2_ sensor response and time response of films made with and without binders were evaluated in the study. This work also investigated the performance of tin dioxide nanoparticles made using a new combustion synthesis approach.

## 2. Experimental

### 2.1. Synthesis of SnO_2_ Nanoparticles

**Figure 1.**
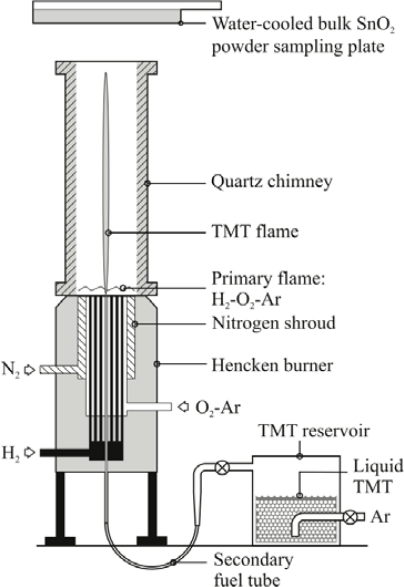
Schematic of the facility used to generate the combustion-synthesized SnO_2_ powders.

The SnO_2_ sensing materials were generated using the University of Michigan combustion synthesis facility that is described in detail in Bakrania *et al.* [17,18] and Miller *et al.* [19]. A schematic of the facility is presented in Figure 1. Briefly, a multi-element diffusion flame burner (a Hencken burner) was used to create the high-temperatures needed for SnO_2_ synthesis using a hydrogen/oxygen (H_2_/O_2_) flame diluted with argon. Liquid tetramethyltin [TMT, (CH_3_)_4_Sn, 98% purity, Alfa Aesar] was used as the precursor for the SnO_2_, and was introduced to the center of the burner by bubbling argon through the TMT reservoir. All gases were a minimum of 99.99% purity (Cryogenic Gases). A quartz chimney and nitrogen co-flow were used to maintain the high temperatures above the burner exit and limit room air entrainment. Temperatures ranged from 1,400 K, near the surface of the burner, to 500 K at a height of 30 cm above the burner. A water-cooled plate (T ∼ 60 °C) was located at a height of 37 cm above the surface of the burner to thermophoretically collect the flame-generated SnO_2_ nanopowders. The average powder sampling rate was measured to be 1.6 g/hr. The SnO_2_ powders were ground using mortar and pestle to fragment large aggregates prior to fabricating the sensor films. The SnO_2_ nanoparticles produced using this approach have been extensively characterized. The average size of the SnO_2_ particles generated for use in this sensor study is reported below. Details on materials analysis studies of SnO_2_ nanoparticles created using this novel method can be found in Bakrania *et al*. [17].

### 2.2. Sensor Fabrication

**Figure 2.**
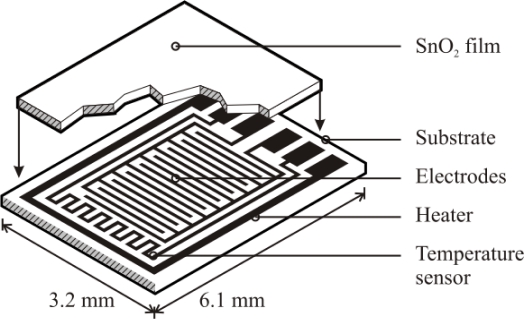
Schematic of the sensor platform (Heraeus MSP 632). The sensing and heating circuits are not drawn to scale.

As noted earlier, two thick film deposition methods were explored: a binder-paste method and a dispersion-drop method. For the binder-paste method, the SnO_2_ pastes were made using either organic- or silicate-based binders. Each sensor was made by depositing the SnO_2_ films onto identical sensor platforms (Heraeus MSP 632) equipped with interdigitated electrodes, heating circuits and temperature sensing circuits. A schematic of the sensing platform is shown in Figure 2. The sensor platforms used platinum electrodes (10 μm electrode separation) deposited on alumina substrates.

Earlier research has shown that binders can provide better film rigidity, and they can improve sensor response by lowering the absolute film resistance [20]. The three binder paste recipes used in this work are listed in Table 1 and were adopted from screen-printing and porous plug fabrication approaches suggested by Lee *et al.* [7] and Ihokura *et al*. [21]. The three recipes are commonly used for binders in thick and thin film sensors [22] and were targeted to identify the binder-film combination that produced high quality and repeatable sensor response without using a screen printer to create the sensor film. The paste application and heat treatment methods are also provided in Table 1. The sintering steps were conducted using a muffle furnace (Fisher Isotemp Basic). Typical SnO_2_ film thickness for the binder-paste methods ranged between 40 to 60 μm.

The dispersion-drop method did not use binders. The powders were dispersed in an ethanol-water solution (15% C_2_H_5_OH in distilled water) using a sonicator (Sonics VC-505 Ultrasonic processor) yielding ∼1.85 wt% SnO_2_ in the dispersion. A micropipetter was used to deposit a 10 μL drop of the dispersion onto a clean sensor platform. Typically, the drop was allowed to evaporate at ambient conditions followed by a low heating step. The low heating step was performed in the muffle furnace at 80 °C for 30 mins. This was followed by another deposition step to add a second layer and a low heating step to yield a film of SnO_2_ (∼5 μm thick per layer). Generally five such layers of tin dioxide were deposited before sintering the film at a higher temperature of 500 °C for 1.5 hrs. The dispersion-drop method yielded total film thicknesses of ∼25 μm.

In order to compare the combustion-generated SnO_2_ powders with commercially available materials, SnO_2_ powder from Alfa Aesar (99.9% metal basis) was also used to prepare a sensor. The commercial SnO_2_ powder was ground using mortar and pestle before applying to a sensor platform using the silicon-based binder recipe and heat treatment described in Table 1.

### 2.3. Sensor Testing

**Figure 3.**
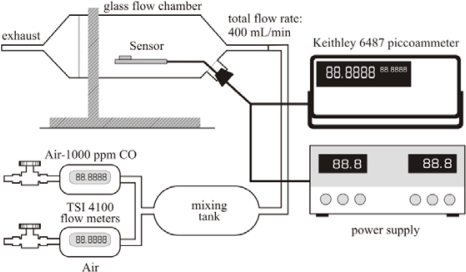
Schematic of the sensor test facility.

The sensor testing facility, shown schematically in Figure 3, consisted of a glass isolation chamber (with approximate volume of 600 mL), the flow manifold for the reference gas (dry air) and the target gas (CO dilute in dry air), the measurement electronics, and the sensor platform. Two digital flow meters (TSI 4100 Series) controlled the flow of gases into a mixing tank upstream of the flow chamber. The sensor tests were performed using dry air and a CO-dry air mixture (1,000 ppm CO, 99% purity, Cryogenic Gases) flowing at a total volumetric rate of 400 mL/min (or 200 mL/min for each component gas). A DC power supply (BK Precision 1760A) was used to power the resistive heater of the sensor. The resistance of the temperature circuit was measured before and after a test while the electrode resistance was measured continuously during the experiments (Keithley 6487 Picoammeter/Voltage Source). Sensing measurements were performed after 24 hrs of conditioning the sensor at an operating temperature of 330 ± 5 °C. The sensor response was defined as *S* = *R_a_*/*R_g_*, where *R_a_* is the resistance in air and *R_g_* is the resistance in target gas, or CO in this case. Time response *τ* was calculated using an algorithm that evaluated the first order time response for the sensor signal. All tests were conducted using a carbon monoxide mole fraction of 500 ppm.

### 2.4. Materials Analysis

Samples of the binder pastes were deposited on glass slides using the same methods used for deposition of the films onto the sensor platforms (including heat treatments). The samples on the glass slides were evaluated using x-ray diffraction (XRD, Scintag Theta-Theta) analysis, scanning electron microscopy (SEM, Philips XL30), and optical microscopy. XRD analysis was used to determine the Scherrer crystallite sizes of the SnO_2_ powders. Scans for phase identification and for average additive particle size were obtained using increments of 0.02° 2θ and CuKα radiation (*λ* = 1.5405 Å). The scans were obtained over a 2θ range of 20°–90° at a scan rate of 5° 2θ/min. Spectral scans for average crystallite size for SnO_2_ were measured over a 2θ range of 22°–31° at a scan rate of 0.5° 2θ/min. Peak positions and relative intensities of the powder patterns were identified by comparison with reference spectra [23]. The average crystallite size was determined from the XRD spectra using the Scherrer equation:

(1) dXRD=0.9λβ12 cos θ

where *d_XRD_* is the average crystallite size, *λ* is the source wavelength, *β_1/2_* is the full-width at half-maximum of the peak used for the analysis and θ is the XRD scattering angle of the peak.

SEM and optical microscopy were used to evaluate film quality. Transmission electron microscopy (TEM, Philips CM-12) was used to determine particle morphology and primary particle size. The TEM samples were prepared by depositing samples of the dispersion-drop mixture onto TEM copper grids (3 mm diameter, Electron Microscopy Sciences, carbon film, 300 mesh copper). The TEM samples were not heat-treated.

## 3. Results and Discussion

The following sections present the results of the effects of the different fabrication methods on sensor performance. All results presented below were obtained using SnO_2_ made via the combustion synthesis process with the exception of the comparison with the commercial SnO_2_ materials.

### 3.1. Materials Characterization of SnO_2_

**Figure 4.**
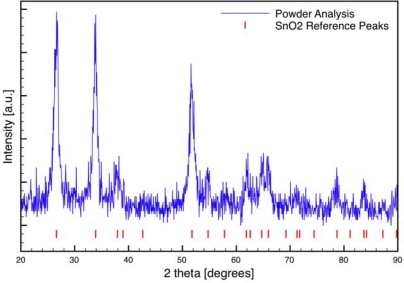
X-ray diffraction analysis of combustion generated tin dioxide nanoparticles. The vertical bars represent the reference peaks for the cassiterite phase of tin dioxide.

**Figure 5.**
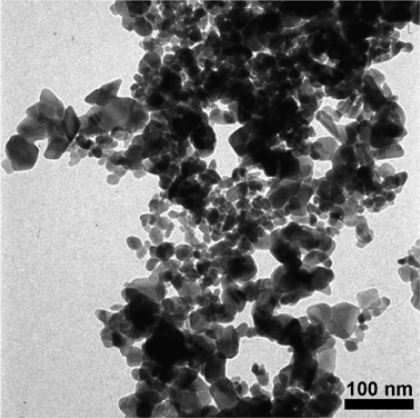
TEM image of SnO_2_ particle morphology.

A full spectrum scan of the sintered combustion synthesized SnO_2_ powder is provided in Figure 4. All peaks indexed to the cassiterite phase of tin dioxide. XRD Scherrer analysis was used to determine if materials restructuring was a concern for interpreting the results of the different sensor fabrication methods. The average crystallite size of the sintered and unsintered SnO_2_ powders was 15 ± 1 nm, regardless of the sintering time (1.5–2 hrs) or temperature considered in the study (500 °C and 600 °C). The XRD results are consistent with TEM imaging of the tin dioxide powders obtained from the dispersion-drop samples. An example of the TEM imaging results is presented in Figure 5. The SnO_2_ particles exhibit dense agglomerate structures with primary particle sizes ranging from 10–40 nm. Based on the XRD and TEM data, the results of this study are not attributed to differences in SnO_2_ particle size or composition for the two categories of films.

### 3.2. Binder-Paste Sensors

**Figure 6.**
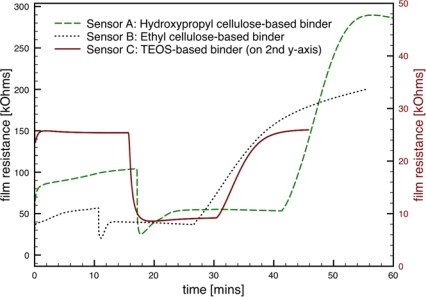
Representative SnO_2_ gas sensor response for sensors made using the three binder recipes. The TEOS-based sensor resistance is plotted on the secondary y-axis to facilitate comparison of the data.

Representative gas sensor response to 500 ppm CO in dry air for each of the binder recipes and processing considered in the study is presented in Figure 6. As shown in the figure, the absolute film resistance for Sensor C was considerably lower than the sensors made using the other binder recipes. The transient behavior observed within the first two minutes of the tests is common to all the sensors, and corresponds to the initial interaction between the film and the voltage applied by the picoammeter to measure the film current [24]. As each sensor is exposed to CO, the sensor responds with a decrease in the film resistance; however, the sensors with cellulose-based binders (Sensors A and B) show “overshoot” in the sensor response before reaching a plateau in the film resistance. Such behavior may be attributed to secondary reactions that take place between the target gas and/or the detection products and the binder material. Additionally, Sensors A and B do not return to the initial resistance values after the flow of CO is stopped. The sensor made with the silicate-based binder (Sensor C) shows preferred sensor behavior, with no “overshoot” and no hysteresis in the baseline resistance.

**Figure 7.**
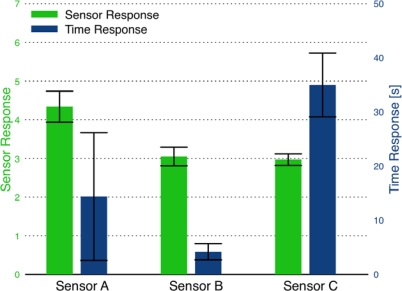
Comparison of sensor response and time response for sensors fabricated using the three binder recipes. Sensor A: hydroxypropyl cellulose-based binder, Sensor B: ethyl cellulose-based binder, and Sensor C: silicate-based binder. The error bars represent the standard deviations for each sensor.

The sensor response and time response for individual sensors prepared using each binder recipe is presented in Figure 7. The error bars in the figure represent the standard deviation in the response of each sensor upon exposure to the target gas. Because sensors A and B do not return to consistent baseline resistances, the *R_a_* value for each sensor response is set as the initial resistance determined at the start of the experiment. Therefore, the error bars represent the variation in the values measured for *R_g_*, and do not account for variation in *R_a_*. The sensor response was fairly consistent between the three binder-paste recipes. The slower time response of Sensor C is offset by the higher quality of the sensor signal. Multiple sensors fabricated using the same binder-paste recipe indicated the reproducibility of the sensor response and time response was poor. For example, multiple sensors produced in the same batch using the same silicate binder-paste as Sensor C yielded responses ranging from *S* = 5 to 20. While such high sensor response is promising in terms of gas sensor performance of the combustion-generated SnO_2_ particles; the results were difficult to replicate.

**Figure 8.**
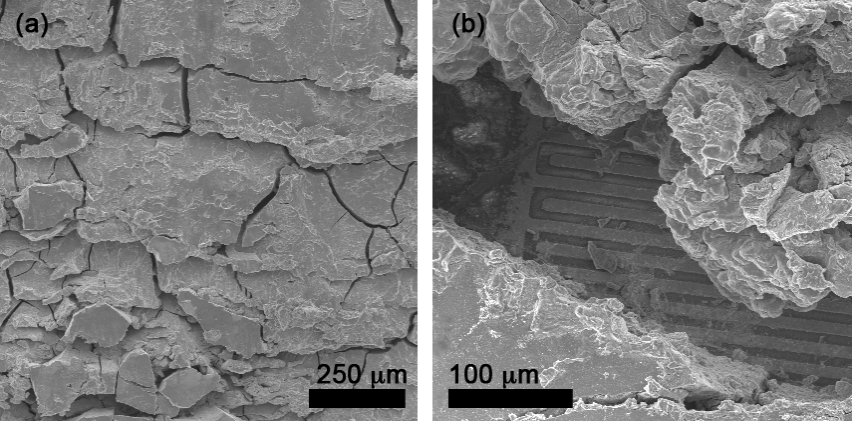
SEM images of tin dioxide gas sensor films deposited using the binder-paste method (Sensor C recipe). Panel a) presents a top view of the film and typical number and size of the film fractures. Panel b) presents a section of the film where the electrodes and the sensor support have been exposed.

There are multiple reasons that can lead to such variability in sensor performance. Even minor variations in film deposition can lead to non-reproducible contact properties between the film and the electrodes. Additionally, since the binder solution was highly volatile (due to the use of ethanol), evaporation during paste processing leads to varying paste rheologies, and therefore varying film properties. As seen in Figure 8, SEM imaging confirms the binder-paste recipe yields fractured films with highly non-uniform microstructures (e.g., film thickness ranging from 30 μm to 60 μm). The irregularity of the binder paste films was exacerbated by the difficulty maintaining consistent rheological properties between pastes. As a consequence, the variability and the high resistance in the sensor performance for binder-paste films is directly attributed to the poor quality of the film structure.

### 3.3. Dispersion-Drop Sensors

**Figure 9.**
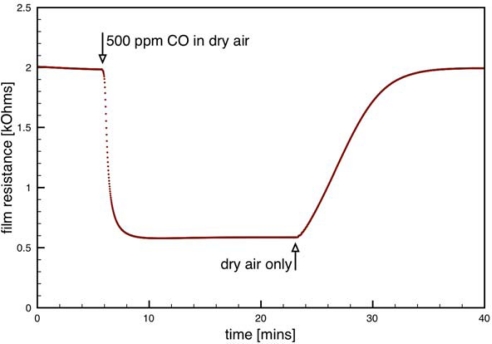
Typical sensor response for a dispersion-drop sensor at an operating temperature of 330 °C.

The dispersion-drop method did not involve binders. Instead, an ethanol-water solution was used to make a dispersion of the SnO_2_ particles. Five dispersion-drop layers, each followed by an annealing step, resulted in an approximately 25 μm thick film. Typical performance of the dispersion-drop sensor is presented in Figure 9. Note the quality of the sensor response to 500 ppm of CO is comparable to that of Sensor C with negligible hysteresis.

**Figure 10.**
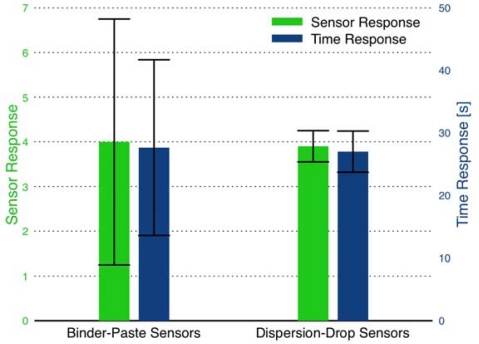
Comparison of sensor response and time response between binder-paste (TEOS-based recipe) and dispersion-drop film deposition methods. The error bars represent the variability in the response of several sensors prepared using each method - in other words, sensor reproducibility.

The sensor response and time response of multiple sensors produced using each of the film deposition methods are compared in Figure 10. The data demonstrate the reproducibility of the binder-paste and the dispersion-drop methods. The bar chart indicates comparable average sensor response for the two deposition methods; however, the dispersion-drop method demonstrates much lower variability between the multiple sensors fabricated using the same method. Additionally, comparing the absolute baseline resistance (in dry air) of the films, the dispersion-drop method yields lower resistances (*R_a_* = 1–10 kΩ) than the films deposited using the binder-paste method (*R_a_* = 100–500 kΩ). This result indicates better electrical contact is achieved using the dispersion-drop method to deposit the tin dioxide films.

**Figure 11.**
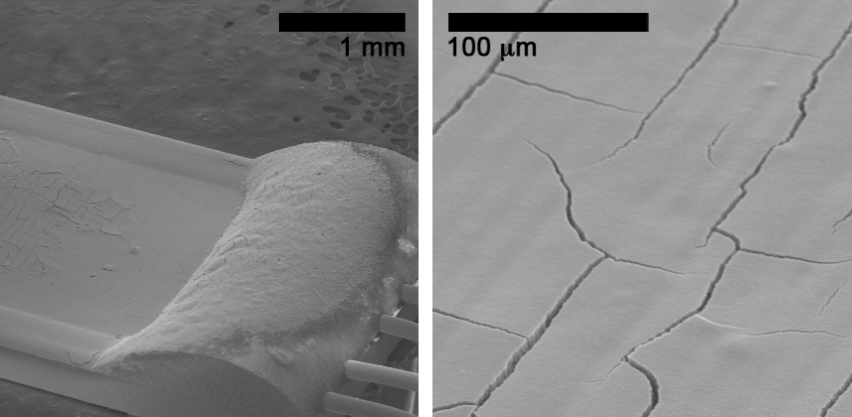
SEM images of a single layer of tin dioxide film deposited using the dispersion-drop method.

SEM imaging of a single layer of tin dioxide deposited using the dispersion-drop method is presented in Figure 11. The images show that much higher film uniformity is achieved using the dispersion-drop method. Although some small fractures are still present in the dispersion-drop films, the extent of the fractures is much less extreme than with the binder-paste films; and the thickness of the dispersion-drop films is also much more consistent.

### 3.4. Further Characterization of the Dispersion-Drop Sensors

Based on the improved repeatability of the performance and the higher quality of the sensors fabricated using the dispersion-drop method, additional studies of the dispersion-drop sensor performance were conducted. We first explored methods to improve the film bonding to the sensing platform and to enhance inter-particle connectivity. A common method of achieving better film bonding is to combine the sensitive powders with either glass frits [11] or aluminum oxide [21,25] in equal quantities. Therefore, a dispersion of SnO_2_ and alumina nanopowder (Al_2_O_3_, Sigma Aldrich) in 1:1 weight ratio was deposited on the sensing platform. Using the standard process, the sensor was tested upon exposure to CO as before. Contrary to the objective, the response during CO exposure was non-ideal with low sensor response (*S* = 1.83 ± 0.27).

As noted earlier, film thickness and porosity can affect sensor performance. The film thickness and porosity are both functions of the number of layers used to create the sensing film. Additional layers can generate a dense network of pores that can alter the response of the sensors to target gases. Becker *et al*. [26], for instance, have shown how thin films (50–300 nm) of tin dioxide sensors are more sensitive to oxidizing species while thick films (15–80 μm) are more sensitive to reducing species as explained by the pore diameters and the resulting depth profiles of the gas species. Karthigeyan and coworkers [27] also demonstrated the dependence of sensitivity on film thickness for thin film sensors (10–55 nm).

**Figure 12.**
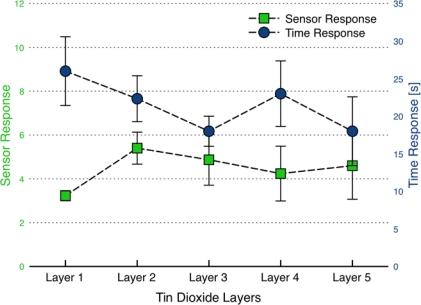
Sensor performance as a function of deposition layer using the dispersion-drop method. Each deposition step was followed by a 500 °C annealing step for 1.5 hrs. The error bars represent standard deviations associated with three tests conducted after each deposition on an individual sensor.

To investigate how film porosity and thickness affect sensor performance, sensor response as a function of addition tin dioxide layers was monitored for the dispersion-drop films. A sintering step (at 500 °C for 1.5 hrs) followed each deposition step instead of the standard procedure where the sintering step followed the deposition of the fifth and final layer only. Figure 12 presents the sensor response and time response measurements as a function of the number of layers deposited on the sensing platform. Each deposited layer was determined to be ∼5 μm thick using side-view SEM images of films. Such tests were conducted with a number of sensors, each yielding results similar to those shown in Figure 12.

Comparing sensor response, there is a marked improvement in sensor response and time response with the addition of the second layer. The addition of subsequent layers had little effect on sensor performance. These observations were repeated with other sensors and are consistent with results by Lee *et al.* [7].

After the first two layers, recall that not only does the sensor consist of two layers which are nominally 10 μm thick (total), the film has received two sintering steps as well as conditioning twice at 330 ± 5 °C. Therefore, the sensor performance may be attributed either to physical layering or to the additional heat treating of the film. In order to understand the effect of repeated heat treating on the film, three sensors with identical SnO_2_ dispersion layers were sintered at 500 °C for 1.5, 3 and 5 hours in the furnace. No discernible differences were observed in sensor response or time response for the sensors. This suggests heat treating is not a dominant influence on the results presented in Figure 12. That leaves the physical addition of the second layer to be the primary driver for the observed improvement in performance. The second layer could provide a denser network of interconnected particles compared to the single film layer [7]. A denser network may not affect diffusion as long as the pore sizes are still sufficiently large for the target gases to freely flow into the film. The second layer of the film then provides more surface area for reaction and response to the target gas. However, if the pore sizes do change dramatically, on the order of the mean free path of molecules in air (∼100 nm), then changes in the film density can have noticeable impact on sensor behavior through both changes in surface area and diffusion transport times.

**Figure 13.**
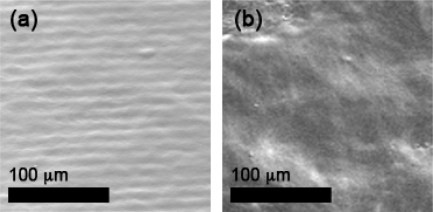
SEM images of the sensor film after drop deposition of (a) the first layer and after drop deposition of (b) the fifth layer. The ridges observed in (a) are a result of the electrodes under the first layer of SnO_2_ film.

SEM images of the surfaces of dispersion-drop films after one layer and five layers have been deposited onto the sensor platform are presented in Figure 13. The images reveal relatively smooth surfaces without the fractures that were typically present for the films deposited without the sintering steps between layer depositions. The absence of fractures is attributed to sintering relieving stresses formed in the film. Since the fractures are much larger than the mean free path of molecules, they may not affect gas-sensor interactions (as suggested by Sakai *et al.* [28]); however, they can influence film bonding. In this study, the layer-by-layer sintering method yielded (after 5 layers) sensor response and time response values of 4.5 and 20 seconds respectively, which is slightly better than the standard dispersion-drop method with the single sintering process, which resulted in an average *S* = 3.7 and *τ* = 26 seconds. Additionally the layer-by-layer deposition yielded consistently low base resistance of *R_a_* ∼ 2.5 kΩ, suggesting better electrical contact between the film and the electrodes. Investigation of pore sizes and density of particle network as a function of film depth is a difficult yet useful approach to understanding how diffusion is affected by layering. Such investigations can provide key insights for the results presented here, but are beyond the scope of this study.

### 3.5. Comparison of Combustion-Generated and Commercial SnO_2_ Powders

**Figure 14.**
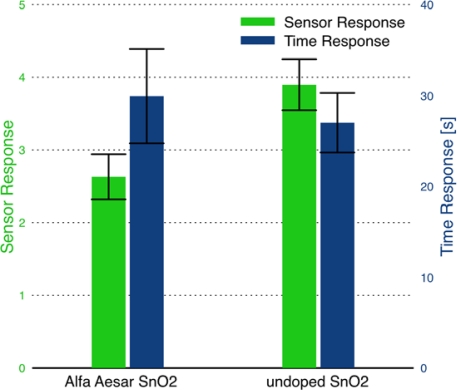
Comparison of sensor performance between combustion generated and commercial tin dioxide powders. The error bars represent the variation in response for multiple sensors.

The performance of sensors made using the combustion-generated SnO_2_ powder was compared to that made using a commercial powder of tin dioxide. The binder-paste deposition method (Sensor C recipe) was used because it was difficult to achieve dispersion with the large particle sizes present in the commercial powders. The sensors were fabricated in parallel on two multi-sensor platforms, each experiencing identical processing steps. A comparison of the sensitivity and time response of the two sensors is provided in Figure 14. The sensor made with combustion generated SnO_2_ demonstrated higher sensitivity by 50% than the commercial powder sensor, while having similar time response. XRD analysis of the powders reveals a significant difference between the Scherrer crystallite sizes. While the average crystallite size for the combustion generated SnO_2_ was 15 ± 1 nm, the Alfa Aesar powder yielded crystallite sizes of ∼55 nm. The results are consistent with expectations of the effect of smaller crystallite size improving sensor performance, as discussed in detail in Yamazoe *et al.* [29].

## 4. Conclusions

The results of the study further demonstrate the strong effects thick-film fabrication methods can have on gas sensor response and the resulting challenge of comparing absolute sensor performance between different fabrication methods and materials. The dispersion-drop sensors yielded excellent repeatability as represented by the low standard deviation for the typical sensor response compared to the binder-paste sensors. Additional processing, such as screen printers, are necessary to reduce the variability in the film microstructural characteristics and the resulting sensor performance for sensors made using binder-paste methods. On the other hand, high quality films, in terms of structure and uniformity and resulting sensor performance, can be achieved with minimal film processing using the new dispersion-drop method demonstrated in this work. Thus, reducing the number of fabrication steps required and the associated costs. The results also show that the combustion-generated SnO_2_ materials yield sensors with good sensor response (*S* = 4) and time response (*τ* = 27 s).

## Figures and Tables

**Table 1.** Binder-paste recipes and film preparation methods.

**Sensor A**	**Sensor B Recipe**	**Sensor C**
0.10 g SnO_2_	0.32 g SnO_2_	3.25 mL TEOS
0.05 g hydroxypropyl cellulose	0.32 g ethyl cellulose	1.35 mL ethanol
6.25 mL isopropyl alcohol	2.87 g α-terpineol soln.	0.35 mL H_2_O
		0.05 mL 4% HCl
		SnO_2_ with 6 wt% silica
**Procedure**		

Sonicated for 20 mins	Mixed and stirred	Made into paste
4 drop-coats with syringe	drop-coated with syringe	applied using spatula
sintering: 2 hrs @ 600 °C	sintering: 2 hrs @ 600 °C	sintering: 2 hrs @ 500 °C

## References

[1] Miller T.A., Bakrania S.D., Perez C., Wooldridge M.S., Geckeler K.E., Rosenberg E. (2006). Nanostructured tin dioxide materials for gas sensor applications. Functional Nanomaterials.

[2] Varghese O.K., Grimes C.A. (2003). Metal oxide nanoarchitectures for environmental sensing. J. Nanosci. Nanotechnol.

[3] Wang C.C., Akbar S.A., Madou M.J. (1998). Ceramic based resistive sensors. J. Electroceram.

[4] Gopal Reddy C.V., Manorama S.V. (2000). Room temperature hydrogen sensor based on SnO_2_:La_2_O_3_. J. Electrochem. Soc.

[5] Barsan N., Schweizer-Berberich M., Göpel W. (1999). Fresenius, fundamental and practical aspects in the design of nanoscaled SnO_2_ gas sensors: a status report. J. Anal. Chem.

[6] Diéguez A., Romano-Rodríguez A., Morante J.R., Kappler J., Bârsan N., Göpel W. (1999). Nanoparticle engineering for gas sensor optimisation: improved sol–gel fabricated nanocrystalline SnO_2_ thick film gas sensor for NO_2_ detection by calcination, catalytic metal introduction and grinding treatments. Sens. Actuat. B Chem.

[7] Lee S., Lee G., Kim J., Kang S.L. (2007). A novel process for fabrication of SnO_2_-based thick film gas sensors. Sens. Actuat. B Chem.

[8] Durrani S.M. (2006). Biasing voltage dependence of sensitivity of electron beam evaporated SnO_2_ thin film CO sensor. Sensors.

[9] Ando M., Tsuchida T., Miura N., Yamazoe N. (1996). Influences of microstructure on hydrogen sulfide sensing characteristics of tin dioxide films. Nippon Kagaku Kaishi.

[10] Hübner H.P., Obermeier E. (1989). Reactively sputtered tin oxide thin-film gas sensors: correlation between fabrication parameters and co-sensitivity. Sens. Actuat.

[11] Garje D., Aiyer R.C. (2006). Electrical and gas-sensing properties of a thick film resistor of nanosized SnO_2_ with variable percentage of permanent binder. Int. J. Appl. Ceram. Technol.

[12] Viricelle J.P., Riviere B., Pijolat C. (2005). Optimization of SnO_2_ screen-printing inks for gas sensor applications. J. Eur. Ceram. Soc.

[13] Ahmad A., Walsh J. (2003). The influence of precursor powders and processing parameters on the properties of SnO_2_-based gas sensors. J. Mater. Sci.

[14] Rue G.H., Lee D.S., Lee D.D. (2004). Effects of substrates on properties of tin oxide gas sensors. Jpn. J. Appl. Phys.

[15] Shimizu Y., Hyodo T., Egashira M. (2004). Mesoporous semiconducting oxides for gas sensor application. J. Eur. Ceram. Soc.

[16] Vaishnav V.S., Patel P.D., Patel N.G. (2005). Preparation and characterization of indium tin oxide thin films for their application as gas sensors. Thin Solid Films.

[17] Bakrania S.D., Miller T.A., Perez C., Wooldridge M.S. (2007). Combustion of multiphase reactants for the synthesis of nanocomposite materials. Combust. Flame.

[18] Bakrania S.D., Perez C., Wooldridge M.S. (2007). Methane-assisted combustion synthesis of nanocomposite tin dioxide materials. Proc. Combust. Inst.

[19] Miller T.A., Bakrania S.D., Perez C., Wooldridge M.S. (2005). A new method for direct preparation of tin dioxide nanocomposite materials. J. Mater. Res.

[20] Yasunaga S., Sunahara S., Ihokura K. (1986). Effects of tetraethyl orthosilicate binder on the characteristics of an SnO_2_ ceramic-type semiconductor gas sensor. Sens. Actuat.

[21] Ihokura K., Watson J. (1994). The Stannic Oxide Gas Sensor - Principles and Applications.

[22] Guidi V., Butturi M.A., Carotta M.C., Cavicchi B., Ferronia M., Malagu C., Martinellia G., Vincenzia D., Sacerdoti M., Zen M. (2002). Gas sensing through thick film technology. Sens. Actuat. B Chem.

[23] JCPDS (1990). Powder Diffraction File.

[24] Watson J., Ihokura K., Coles G.S.V. (1994). The tin dioxide gas sensor. Meas. Sci. Technol.

[25] Saha M., Banerjee A., Halder A.K., Mondal J., Sen A., Maiti H.S. (2001). Effect of alumina addition on methane sensitivity of tin dioxide thick films. Sens. Actuat. B Chem.

[26] Becker T., Ahlers S., Bosch-v.Braunmühl C., Müller G., Kiesewetter O. (2001). Gas sensing properties of thin- and thick-film tin-oxide materials. Sens. Actuat. B Chem.

[27] Karthigeyan A., Gupta R.P., Burgmair M., Sharma S.K., Eisele I. (2002). Influence of oxidation temperature, film thickness and substrate on NO_2_ sensing of SnO_2_ ultra thin films. Sens. Actuat. B Chem.

[28] Sakai G., Baik N.S., Miura N., Yamazoe N. (2001). Gas sensing properties of tin oxide thin films fabricated from hydrothermally treated nanoparticles: Dependence of CO and H_2_ response on film thickness. Sens. Actuat. B Chem.

[29] Yamazoe N. (1991). New approaches for improving semiconductor gas sensors. Sens. Actuat. B Chem.

